# Serum microRNAs in osteoporotic fracture and osteoarthritis: a genetic and functional study

**DOI:** 10.1038/s41598-021-98789-w

**Published:** 2021-09-29

**Authors:** Clara Pertusa, Juan J. Tarín, Antonio Cano, Miguel Ángel García-Pérez, Damián Mifsut

**Affiliations:** 1 Research Unit, INCLIVA Health Research Institute, 46010 Valencia, Spain; 2 Department of Cellular Biology, Functional Biology and Physical Anthropology, University of Valencia, 46100 Burjassot, Spain; 3 Department of Pediatrics, Obstetrics and Gynecology, University of Valencia, 46010 Valencia, Spain; 4 Department of Genetics, University of Valencia, 46100 Burjassot, Spain; 5 Orthopedic Surgery and Traumatology, Clinic Hospital, INCLIVA Institute of Health Research, 46010 Valencia, Spain

**Keywords:** Bone, Cartilage, Epigenetics, Genetic markers, Biomarkers

## Abstract

The rising incidence of bone pathologies such as osteoporosis and osteoarthritis is negatively affecting the functional status of millions of patients worldwide. The genetic component of these multifactorial pathologies is far from being fully understood, but in recent years several epigenetic mechanisms involved in the pathophysiology of these bone diseases have been identified. The aim of the present study was to compare the serum expression of four miRNAs in women with hip fragility fracture (OF group), osteoarthritis requiring hip replacement (OA group) and control women (Ctrl group). Serum expression of miR-497-5p, miR-155-5p, miR-423-5p and miR-365-3p was determined in a sample of 23 OA women, 25 OF women and 52 Ctrl women. Data shown that women with bone pathologies have higher expression of miR-497 and miR-423 and lower expression of miR-155 and miR-365 than control subjects. Most importantly, miR-497 was identified as an excellent discriminator between OA group and control group (AUC: 0.89, *p* < 0.000) and acceptable in distinguishing from the OF group (AUC: 0.76, *p* = 0.002). Our data suggest that circulating miR-497 may represent a significant biomarker of OA, a promising finding that could contribute towards future early-stage diagnosis of this disease. Further studies are required to establish the role of miR-155, miR-423 and miR-365 in bone pathologies.

## Introduction

The worldwide rise in life expectancy over the last century has brought increased incidence of late onset diseases, with adverse consequences for patient quality of life. Bone diseases form an important subset of these conditions. With sequelae ranging from pain and deformity to fractures, they dramatically affect patient functional status and can thus trigger significant rapid decline in physical and mental health^1^.

Osteoporosis (OTP) is a multifactorial disease characterized by low bone mass and microarchitectural deterioration of the bone, whose main consequence is bone fracture (BF) due to fragility. OTP causes more than 8.9 million BF each year worldwide, and its prevalence is rising in parallel with growing longevity, heralding an increasingly important role for BF prevention in future years^2^.

Another multifactorial bone disease is osteoarthritis (OA). Characterized by joint pain and functional impairment, it can result in pathological changes such as destruction of articular cartilage, thickening of the subchondral bone and osteophyte formation. OA affects more than 40 million people across Europe, and with the increase in life expectancy it is predicted to become one of the main causes of disability worldwide^3^.

Both diseases have greater prevalence in women than men, whereas risk factors shared by both include age and genetic predisposition^4^. Although the genetics of both diseases are complex, family studies have reported high heritability for bone phenotypes. However, genetic variants alone can account for only a small portion of disease heritability. To shed more light this, the role of epigenetics in these pathologies has been brought into greater focus in recent years^5^.

Epigenetic mechanisms allow the transmission of phenotypic changes without altering the DNA sequence. Several of these mechanisms, such as DNA methylation, have already been linked to development of OTP and OA^6^. Another epigenetic marker gaining importance in OTP and OA research are miRNAs, small non-coding RNAs which negatively regulate gene expression, and are involved in normal biological functions by post-transcriptional regulation of gene expression. Each miRNA can have multiple mRNA targets and a gene can be regulated by multiple miRNAs. miRNAs are endogenously synthesized, but are present in many bio-fluids outside the cell (circulating miRNAs) and are remarkably stable^7^. Many recent studies have explored the association between alterations in circulating miRNA levels and pathological conditions in organisms, revealing their potential as diagnostic and prognostic biomarkers, especially in cancer.

miRNAs are known to regulate bone metabolism by interfering in bone remodeling and bone cell differentiation, growth and function^8^. Their altered expression in pathological bone conditions points to the potential for discovering miRNAs that can act as biomarkers for bone disease diagnosis and progression. Effectively, several miRNAs have been proposed as potential biomarkers for OTP, targeting bone homeostasis or the estrogen pathway^9^. In the case of OA, many studies have been made to show the importance of miRNAs in regulating catabolic and anabolic genes, both involved in the development of OA, by targeting upstream signaling pathways or epigenetic factors^10^. These biological functions regulated by miRNAs include chondrocyte proliferation and apoptosis, extracellular matrix metabolism or inflammation^11^. This marks these miRNAs as potential therapeutic targets as well, and RNA sequencing studies providing the whole miRNA-mRNA interactome will be crucial towards that step^12^.

The aim of the present study is to validate four serum miRNAs as possible biomarkers of bone pathologies such as hip OF and OA. This work is the continuation of a previous study conducted by this group^13^ in which 179 serum miRNAs were analyzed in a case–control study. The four miRNAs have been chosen due to significant expression changes observed. Our previous study already validated miR-122-5p, miR-125b-5p and miR-21-5p as valuable biomarkers, but we have chosen not to include them in the present study because there is already strong evidence for these miRNAs as therapeutic targets and as biomarkers for osteoporosis^14–16^. Additionally, we have improved the design of the previous study by including a control group of women without bone pathologies, which, in our opinion, allows us to obtain more solid conclusions.

## Materials and methods

### Study subjects

The study cohort comprised 100 women of Caucasian ethnicity living in Valencia, Spain, recruited consecutively from the Orthopedic Surgery and Traumatology service of the Hospital Clínico and from the Menopause Units of the Hospital Clínico and the Hospital Doctor Peset.

The cohort was stratified into 25 patients with osteoporotic subcapital hip fracture (Fracture group, OF); 23 patients without osteoporosis but with severe (grade 4) hip osteoarthritis according to Kellgren–Lawrence radiological scale^17^ that requiring prosthetic hip placement (Osteoarthritis group, OA); and 52 women without bone pathology (Control group, Ctrl). Women were determined as controls by a self-report, their medical history, and the examinations of the clinicians of our service. Exclusion criteria were history of bone disease other than primary osteoporosis or osteoarthritis, fractures due to high-energy trauma, hyperparathyroidism, hyperthyroidism, renal insufficiency, primary amenorrhea, ongoing cancer treatment and age under 50 years.

This study was approved by the Medical Research Ethics Committee (CEIm) of the Hospital Clínico Universitario of Valencia in accordance with the principles of the Declaration of Helsinki. All participants read and signed informed consent in accordance with INCLIVA Health Research Institute guidelines.

### Biochemical and anthropometric measurements

Blood samples were extracted from participants to obtain serum, which was stored at – 80 °C until use^13,18^. Levels of carboxy-terminal telopeptides of type I collagen (CTx) and 25-Hydroxycholecalciferol (25(OH)D3) were measured by electrochemiluminescence (E170 Modular Analyzer, Roche Diagnostics, Mannheim, Germany). Levels of total alkaline phosphatase (ALP) were determined using an autoanalyzer (Olympus 5400, Tokyo, Japan).

Body mass index (BMI) was calculated as weight (kg) divided by height in square meters (m^2^). Bone mineral density (BMD, g/cm^2^) was quantified using dual energy X-ray absorptiometry (DXA) at the non-dominant proximal femoral neck (FN-BMD), unless this was the fractured hip, and at the lumbar spine (L2–L4, LS-BMD) using a Lunar DPX densitometer (GE Lunar Corporation, Madison, WI, USA), a Norland XR-36 (Norland Medical Systems Inc; Fort Atkinson, WI, USA), or Hologic (Hologic Explorer, Marlborough, MA, USA) densitometers. Standardized BMD (sBMD) was calculated for comparison between subjects^19,20^.

### miRNA isolation

miRNA extraction was performed for each serum sample using the miRNeasy Serum/Plasma Advanced Kit (Qiagen, Hilden, Germany) following the manufacturer’s instructions. As a first step, synthetic RNA Cel-miR-39 (Qiagen) from *C. elegans* was added in the quantity recommended by the manufacturer as a quality control to determine the efficiency of the extraction and for miRNA normalization because of the absence of homologous sequences in humans, with 1 μg of bacteriophage MS2 RNA (Roche, Basel, Switzerland) also added to each sample to act as a carrier.

Briefly, serum samples were thawed and centrifuged for 5 min at 3000×*g*; 200 μl of the supernatant were used for miRNA extraction and afterwards Cel-miR-39 and MS2 RNAs were added. Subsequently, extraction was performed following the manufacturer’s instructions. Total miRNA was eluted in 40 μl H_2_O and stored at – 80 °C until use.

### Cell culture, osteogenic differentiation, and RNA extraction

Human primary osteogenic sarcoma cell line Saos-2 (ATCC, Manassas, VA, USA) cells were cultured in McCoy's 5a Modified medium supplemented with 15% fetal bovine serum (FBS, ThermoFisher, Waltham, MA, USA) and 1% penicillin and streptomycin (ThermoFisher) in humidified air containing 5% CO_2_ at 37 °C. For osteogenic differentiation induction, cells were cultured in 6-well plates until 80% confluency, then switched to osteogenic medium containing 50 μΜ l-ascorbic acid, 10 nM dexamethasone and 10 mM beta-glycerophosphate (Merck). Osteogenic medium was changed twice weekly. Control cells were cultured in equal sized plates with regular medium, changed at the same intervals as the osteogenic medium.

Total RNA, including miRNA, was extracted from the cell culture using TRIzol Reagent (ThermoFisher) following the manufacturer’s instructions. Two biological replicates (with three technical replicates) were used. Briefly, adherent cells were washed with PBS and then lysed by adding 1 ml TRIzol to each well. The lysate was incubated for 5 min at room temperature and 200 μl chloroform was added. After shaking and centrifugation, the clear phase was transferred to a clean tube and 500 μl 2-propanol was added. Samples were incubated at – 20 °C overnight and centrifuged for 10 min at 12,000×*g*. Pellet was washed with 1 ml of 75% ethanol, resuspended in DEPC water and RNA was stored at – 20 °C until further use.

### Analysis of differential expression

Once miRNAs had been extracted from all serum samples, the four selected miRNAs (Hsa-miR-497-5p, Hsa-miR-155-5p, Hsa-miR-423-5p and Hsa-miR-365-3p), as well as the housekeeping (Hsa-miR-93-5p) and Cel-miR-39, were determined using RT-qPCR. Hsa-miR-93-5p was chosen as housekeeping for its widely regarded potential as a reference gene^21,22^ and because in our previous study it was revealed to be a reference miRNA candidate by the GeNorm algorithm in the profiling stage for our sample pool^13^. We used 2 μl of each miRNA sample for retro-transcription (RT) in 6 μl of total volume reaction using the TaqMan Micro RNA Reverse Transcription kit (ThermoFisher) and specific primers for each miRNA contained in the TaqMan miRNA Assay kit (ThermoFisher). Amplification of the RT product was performed using TaqMan Universal PCR Master Mix II (ThermoFisher) and the specific primers and probe for each miRNA present in the TaqMan miRNA Assay kit (ThermoFisher). The reaction was carried out using QuantStudio 5 Real-Time PCR System (ThermoFisher) in 384-well plates, and amplification curves were analyzed with QuantStudio Design and Analysis Software (v1.5.1; ThermoFisher).

### Data treatment and statistical analysis

Normalization of miRNA data was performed as described in^23^. Briefly, a relative quantification (RQ) value to Cel-miR-39 was calculated for every miRNA and transformed to linear scale. After that, normalized relative quantification (NRQ) values were calculated for each miRNA by dividing these RQ by the normalization factor (NF), which is the geometric mean of the RQs of all miRNAs in that sample.

Non-detected target values were set to the detection limit (40 Cts) and then normalized like the other samples, following the guidelines of data analysis software such as DataAssist v3.0 Software (Applied Biosystems) and Real-Time StatMiner Software (v4.0; Integromics, Granada, Spain)^24^.

To minimize the influence of outliers (detected by Tukey’s test), quantitative variables were winsorized (i.e., the data point was replaced with the next highest or lowest non-outlier value). We assumed missing at random, but to adjust for potential bias associated with quantitative missing data, five imputations were run and the average of these was used for statistical analyses.

Fixed-effects analysis of variance (ANOVA) designs were used to compare means between groups. If the ANOVA test was significant, Bonferroni test (when the variances were assumed to be equal) or Dunnett’s T3 test (when the variances were assumed to be unequal) was applied to perform post hoc pairwise comparisons at α = 0.05 level. Levene’s test was used to test the homogeneity of variance for each dependent variable across all level combinations of between-subject factors. Analysis of covariance (ANCOVA) was used to examine differences in the dependent variable (miRNA levels) between groups after adjustment for age and BMI covariates.

Receiver operating characteristic (ROC) curves were established to discriminate patients from controls. The area under the ROC curve (AUC) was considered as a diagnostic value.

The present study shows a statistical power of 97.8% and 99.1% when comparing the OA group versus the Control and OF groups, respectively, to detect a difference of 0.15 in the mean values of miR-497 (less than that detected). This has been estimated assuming the real sample size and ratio between groups, the standard deviations that appear in Table 2 and with a confidence level of 95%. For the analysis, the online Epidat 3.1 software package (http://www.sergas.es/) was used.

All analyses were two-tailed, and statistical significance was defined as *p* < 0.05. Data was analyzed using IBM SPSS statistics for Windows (v.26.0; Armonk, NY: IBM Corp.).

## Results

Anthropometric, biochemical and bone characteristics of the study participants are shown in Table 1. Women in the OF group were significantly older than those in the Ctrl or OA groups. There were no between-group differences in BMI (*p* = 0.877) or LS-BMD related parameters, although the OF group showed a clear trend towards lower LS-BMD than the OA group (*p* = 0.057). However, as expected, there were major differences between groups with respect to FN-BMD, with subjects in the OF group showing worse bone parameters than those in the Ctrl and OA groups. No significant differences between Ctrl and OA women was found in BMD at any skeletal site. Regarding bone metabolism markers, the OF group showed higher levels of CTx than any other group, and both OF and OA groups presented lower vitamin D levels than the Ctrl group (Table 1).

**Table 1:** Anthropometric, biochemical and bone characteristics of the study cohort.

	Control (N = 52)	Osteoarthritis (N = 23)	Fracture (N = 25)	ANOVA *p *value
Age (years)	65.5 ± 6.8	69.9 ± 9.7	76.8 ± 8.3 (a,b)	< 0.0000
Weight (kg)	69.4 ± 11.7	68.7 ± 11.1	68.0 ± 10.6	0.861
Height (cm)	157.5 ± 6.6	157.9 ± 5.1	156.1 ± 4.0	0.495
BMI (kg/m^2^)	28.1 ± 4.9	27.5 ± 3.9	27.9 ± 4.1	0.877
FN-BMD (g/cm^2^)	0.780 ± 0.162	0.800 ± 0.141	0.640 ± 0.122 (b,c)	0.0002
FN T-score	− 1.175 ± 1.442	− 0.313 ± 0.996	− 2.457 ± 1.075 (c,d)	0.00001
FN Z-score	0.144 ± 1.431	0.873 ± 1.061	− 0.795 ± 0.770 (c,d)	0.0005
LS-BMD (g/cm^2^)	0.992 ± 0.170	1.033 ± 0.154	0.922 ± 0.149	0.055
LS T-score	− 1.216 ± 1.445	− 0.585 ± 1.455	− 1.794 ± 1.701	0.097
LS Z-score	0.377 ± 1.577	0.954 ± 1.472	0.294 ± 1.676	0.455
CTx (ng/ml)	0.367 ± 0.153	0.443 ± 0.156	0.659 ± 0.220 (a,b)	< 0.0000
Total-ALP (U/l)	170.7 ± 39.9	144.6 ± 73.0	176.8 ± 92.1	0.176
25(OH)D3 (ng/ml)	25.9 ± 9.7	16.5 ± 9.2 (**c**)	14.4 ± 10.9 (e)	< 0.0000

*BMI* body mass index, *BMD* bone mineral density, *FN* femoral neck, *LS* lumbar spine, *CTx* carboxy-terminal telopeptides of collagen I, *ALP* alkaline phosphatase, *25(OH)D3* 25-hydroxycholecalciferol.

a: p < 0.0000 vs. control.

b: p < 0.01 vs. osteoarthritis.

c: p < 0.01 vs. control.

d: p < 0.0001 vs. osteoarthritis.

e: p < 0.0001 vs. control.

**Figure 1:**
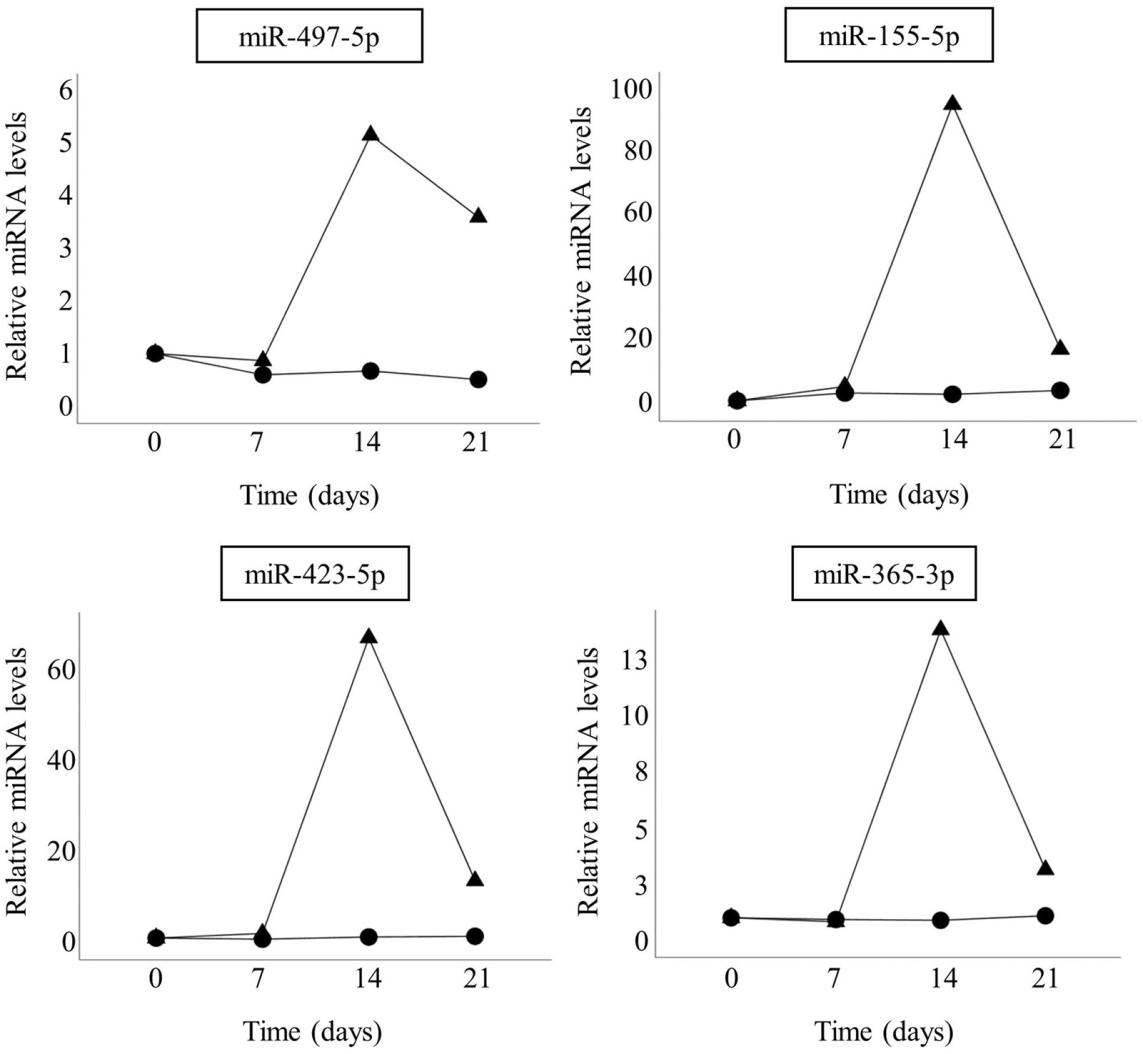
Expression pattern of miR-497-5p, miR-155-5p, miR423-5p and miR-365-3p in Saos-2 cells over 21 days with (▲) or without (●) osteoblastic differentiation induction.

Figure 1 shows that all the miRNAs studied herein have increased expression levels compared to untreated cells during osteogenic differentiation of Saos-2 cells, all with peak expression at 14 days; miR-155-5p and miR-423-5p showed the largest relative increase.

Table 2 shows the average serum expression levels of the miRNAs analyzed in the present study for each group and the *p* values achieved from the ANOVA test. As can be observed, there were significant between-group differences for the four miRNAs studied, even after correction for age and BMI in an ANCOVA analysis (not shown). Significantly lower levels of miR-155 and miR-365 were detected in the bone pathology groups than in controls, although the OA group showed a trend (*p* = 0.082) towards lower levels of miR-365 compared with the OF group. Regarding miR-423, women in the OF group showed higher levels than those in the Ctrl group, while a trend (*p* = 0.066) towards higher levels in OA group women than controls was also observed. Finally, participants with bone pathologies showed higher levels of miR-497 than those in the Ctrl group, although subjects with bone pathologies also differed between each other, showing significantly higher levels of miR-497 in the OA group than the OF group (*p* < 0.01).

**Table 2:** Mean of expression levels for assayed miRNAs.

	Control (N = 52)	Osteoarthritis (N = 23)		Fracture (N = 25)		ANOVA *p *value
	Expression levels	Expression levels	Fold change	Expression levels	Fold change	
Hsa-miR-155	1.93 ± 1.43	0.76 ± 0.45 (a)	− 2.54	0.94 ± 0.58 (**b**)	− 2.05	0.00002
Hsa-miR-365	0.83 ± 0.61	0.31 ± 0.16 (a)	− 2.68	0.47 ± 0.31 (**c**)	− 1.77	0.00004
Hsa-miR-423	1.25 ± 0.98	1.97 ± 1.28	1.58	2.54 ± 1.69 (**c**)	2.03	0.0002
Hsa-miR-497	0.11 ± 0.08	0.38 ± 0.24 (d,e)	3.45	0.18 ± 0.12 (**f**)	1.64	< 0.0000

a: p < 0.0000 vs. control.

b: p < 0.001 vs. control.

c: p < 0.01 vs. control.

d: p < 0.0001 vs. control.

e: p < 0.01 vs. fracture.

f: p < 0.05 vs. control.

**Figure 2:**
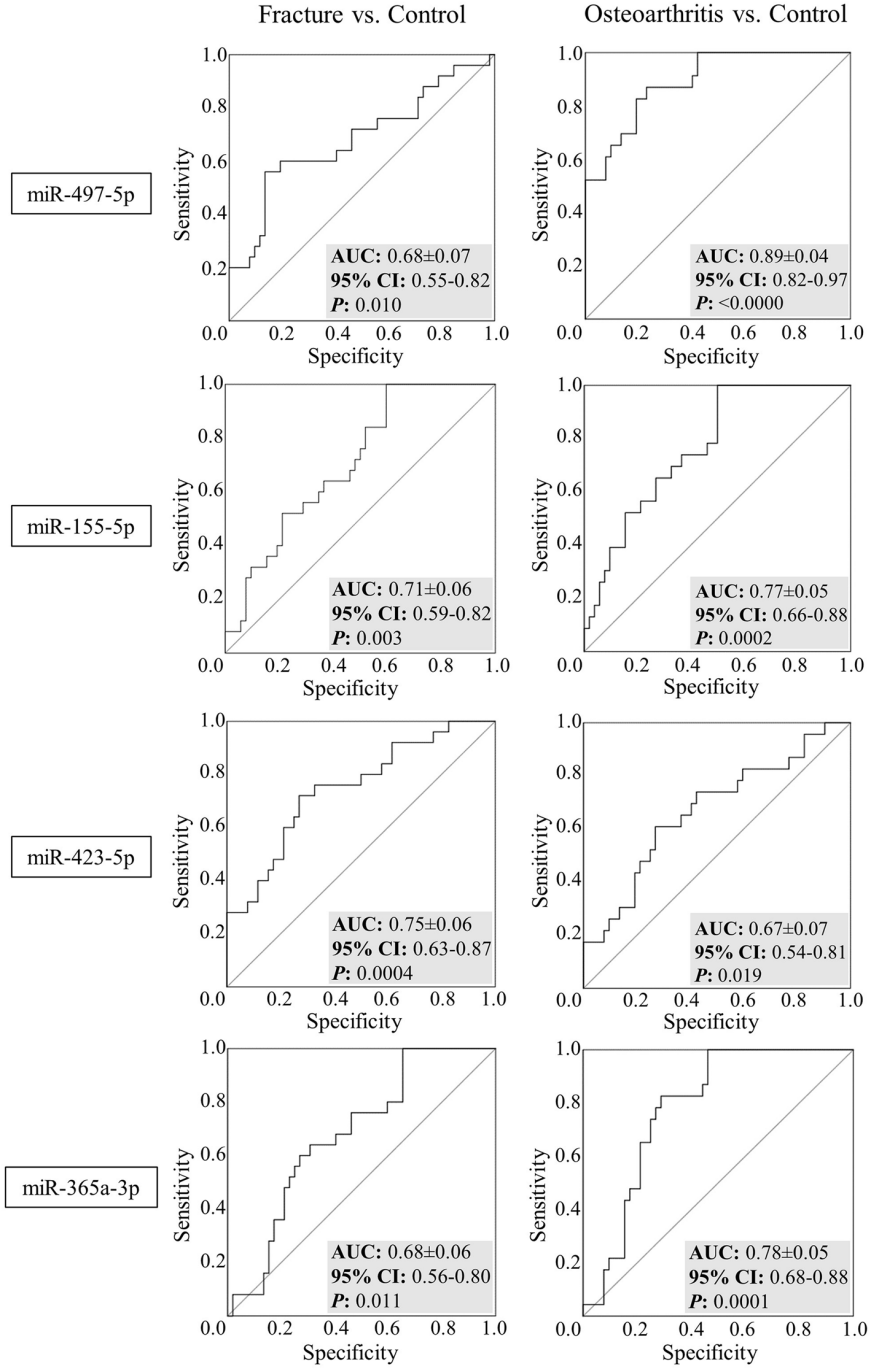
Receiver operating characteristic (ROC) for miR-497-5p, miR-155-5p, miR-423-5p and miR-365-3p, showing diagnostic ability for osteoarthritis and osteoporotic fracture. Area under curve (AUC), confidence intervals (95% CI) and *P*-values are shown.

ROC analysis was performed to evaluate the diagnostic value of these serum miRNAs (Fig. 2). Results showed AUC values between 0.7 and 0.8 for several miRNAs, indicating acceptable discriminative power between patients and controls^25^. Thus, miR-155 proved able to distinguish the OF group (AUC: 0.708, *p* = 0.003) and the OA group (AUC: 0.769, *p* = 0.0002) from the Ctrl group. miR-423 correctly differentiated the OF group from the Ctrl (AUC: 0.748, *p* = 0.0004), while miR-365 was able to discern between the OA and Ctrl groups (AUC: 0.783, *p* = 0.0001). Nevertheless, the miRNA exhibiting most diagnostic power was miR-497 (Fig. 2), which allowed excellent discrimination between the OA and Ctrl groups (AUC: 0.894, *p* < 0.0000), but also an acceptable discrimination between OA and OF groups (AUC: 0.757, *p* = 0.002; not shown)^25^.

## Discussion

In this study we compared the serum levels of four miRNAs in women with bone pathologies (OF and OA) with their levels in control subjects to identify new biomarkers for these diseases. The results showed that miR-497 was able to differentiate the OA group from the remaining groups, emerging as an excellent, specific biomarker for this pathology against the Ctrl group, as indicated by the area under the ROC curve (AUC ≥ 0.8 and < 0.9)^25^. The other miRNAs studied reached an acceptable level of discrimination (AUC ≥ 0.7 and < 0.8) according to Hosmer et al.^25^: miR-155 was able to discriminate both OA and OF groups from the Ctrl group, while miR-423 distinguished the OF group from the Ctrl group and miR-365 discriminated the OA group from the Ctrl group (Fig. 2).

As in the present study, Smith et al.^26^ demonstrated a peak in miR-365 expression in B6 and C3H stromal cells at week 3–4 of osteoblastic differentiation. The role of miR-365 as an inducer of osteoblast differentiation has already been suggested, via the targeting of Histone Deacetilase 4 (HDAC4), which inhibits osteoblast differentiation transcription factor Runx2^27^. miR-155 has previously been described as an inhibitor of osteogenic differentiation through the inhibition of Bone Morphogenic Proteins (BMPs)^28^, which would differ with our results. However, Eguchi et al.^29^, described a peak in miR-155 expression in KUSA-A1 cells 4 h after induction. The increase of miR-497 expression with osteogenesis was also observed by Ma et al.^30^, who described a peak in miR-497 expression in MC3T3-E1 cells 7 days after induction, and Liu et al.^31^ hypothesized that miR-497 could induce osteogenic differentiation via the Smad-signaling pathway, since it targets Smurf2, an inhibitor of this route. However, Grünhagen et al.^32^ observed that although the expression of the miR-497~195 cluster is upregulated during in vitro osteoblastic differentiation, this cluster microRNAs can act as antagonists of BMP signaling in bone cells. About the discrepancies in the peak day among studies, we mainly attribute this to the cell type used, although it may also be due to the use of different osteogenesis-inducing stimulus. Finally, to our knowledge, we are the first to determine miR-423 expression during osteoblast differentiation.

In our previous work^13^, we described increased serum miR-497 levels in women with osteoarthritis compared to those with osteoporotic hip fracture. The present study confirms and amplifies this finding, as we also demonstrate higher miRNA levels in the OA group than in the Ctrl group of women, showing excellent discriminatory power (AUC: 0.894, *p* < 0.000)^25^.

Alterations in miR-497 have been mainly reported in different types of cancer^33^. However, altered levels of miR-497 have also been reported in metabolic syndrome^34^, in axial spondyloarthropathy HLA-B27 + patients^35^, in Parkinson's disease^36^, in mitochondrial muscular dystrophies^37^, in pulmonary fibrogenesis^38^, and in acute cerebral infarction^39^. In bone, overexpression was found to promote differentiation and mineralization of osteoblasts through the JNK signaling pathway^40^, and more specifically through the TGF-beta1/Smads signaling pathway^41^. These studies describe lower levels of miR-497 in osteoporotic bone tissue^30,40,41^, although Ma et al.^30^ also describe lower serum levels of this miRNA in postmenopausal than premenopausal women, albeit without detecting significant differences between postmenopausal women with osteopenia or osteoporosis. Hou et al.^42^ found downregulated miR-497 levels in human osteoarthritic cartilage.

In the present study, circulating miR-497 was upregulated in the OA group compared to the Ctrl group, but also with respect to the OF group, singling it out as an excellent biomarker to discriminate between groups in the former case and an acceptable one in the latter case^25^. However, no explanation was found for the higher circulating levels of miR-497 in the bone pathology groups than the Ctrl group, contrasting with the previously described downregulation in osteoporotic bone tissue samples^30,40,41^ and in OA cartilage^42^. This discrepancy between circulating and tissue miRNA changes has already been described^43,44^ and although it has been attributed to cell mechanisms that can selectively release or retain specific miRNAs^45^, cell damage or increased apoptosis could underlie this discordance^46^.

With respect to the other miRNAs, the present study reveals decreased miR-155 in both pathologies compared to the Ctrl group. Our results concur with previously published findings on skeletal fractures in postmenopausal women^47^, although they differ from those of Adamo et al.^48^, who found increased miR-155 in OA cartilage. However, given that our study was performed on serum samples, the two findings may not be comparable. In this regard, a trend towards higher levels of miR-155 in plasma of controls than OA patients has been observed^49^.

A similar pattern between groups was detected in miR-365, which showed decreased levels in the bone pathology groups compared to the control group. Along these lines, a decrease in the serum level of miR-365 has previously been reported in low-trauma fractures^50^ and in OA patients, although in the latter case the tissue analyzed was cartilage^51^.

Finally, miR-423 is a negative regulator of osteoblastogenesis^52^ and could have an osteogenic function in response to biomaterials^53^. The elevated miR-423 levels in OF (*p* < 0.01) and OA groups (*p* = 0.066) compared to the Crtl group could be compatible with a lower rate of bone formation in these pathologies. Indeed, lower miR-423 levels have been described in osteoporotic bone tissue^54^ in accordance with our data and with its role as a negative regulator of osteoblastogenesis. The exact cause of increased expression of miR-423 during Saos-2 cell differentiation (Fig. 1) remains unclear, although a miR-423-5p mimic has been reported to promote proliferation and inhibit apoptosis in cultured bone marrow mesenchymal stem cells from orofacial bone, while an antisense inhibitor of this miRNA had the opposite effect^55^.

Aside from its strengths, the present study has several limitations, mainly the modest sample size. Additionally, there were significant between-group differences in terms of age. As we studied only women, it is unknown whether our findings can be extrapolated to men, and data was lacking on the lifestyle of the participating subjects, such as dietary habits or physical activity. Finally, it is possible that we have enrolled some women with OA in the control group, but in any case, they would belong to grade 1 according to Kellgren–Lawrence radiological scale^17^, where there are no radiological signs, or these are doubtful.

As a conclusion, in the present study we report that women with OA show higher serum levels of miR-497 than control and OF women, pinpointing this miRNA for its excellent and acceptable discriminatory power against these respective groups. Furthermore, miR-365 and miR-155 are decreased while miR-423 is increased in the bone pathology groups compared with the Ctrl group. This forms a basis for further research into these promising potential biomarkers which could contribute towards future early-stage diagnosis of bone diseases.

## Data Availability

Restrictions apply to the availability of data generated or analyzed during this study to preserve patient confidentiality. However, upon reasoned request to the corresponding author and the Ethics Committee, this data may be transferred to interested researchers if the Ethics Committee, based on this reasoned request, gives its approval.
